# Relevance of IGFBP2 proteolysis in glioma and contribution of the extracellular protease ADAMTS1

**DOI:** 10.18632/oncotarget.2009

**Published:** 2014-05-26

**Authors:** Estefanía Martino-Echarri, Rubén Fernández-Rodríguez, Joan Josep Bech-Serra, María del Carmen Plaza-Calonge, Noemi Vidal, Carmen Casal, Nuria Colomé, Joan Seoane, Francesc Canals, Juan Carlos Rodríguez-Manzaneque

**Affiliations:** ^1^ GENYO. Centre for Genomics and Oncological Research: Pfizer/Universidad de Granada/Junta de Andalucía, Granada, Spain; ^2^ Vall d'Hebron Institute of Oncology (VHIO), Vall d'Hebron University Hospital, Barcelona, Spain; ^3^ Institut de Neuropatologia, Hospital Universitari de Bellvitge, Hospitalet de Llobregat, Spain; ^4^ Institució Catalana de Recerca I Estudis Avançats (ICREA), Barcelona, Spain

**Keywords:** ADAMTS, extracellular proteolysis, glioma, IGFBP2, proteomics

## Abstract

Expression of IGFBP2 (*Insulin-like Growth Factor Binding Protein 2*) has been positively correlated with glioma progression. Although the proteolysis of IGFBP2 has been widely recognized, with consequences as a major modulator of IGFII signaling, the relevance of this post-translational modification has not been well studied in tumors. Using an *in vivo* proteomic approach by Isotope-Coded Protein Label (ICPL), we identified IGFBP2 as a target of the extracellular protease ADAMTS1 (*A Disintegrin And Metalloproteinase with ThromboSpondin motifs 1*). Notably, the proteolytic pattern of IGFBP2 was also detected in human glioma culture cells and, more importantly, in all glioma samples evaluated. In addition, high expression of ADAMTS1 correlates with higher levels of cleaved IGFBP2 in glioblastoma multiforme cases. Using gene expression public databases, we confirmed that IGFBP2 is a poor prognosis marker for gliomas, and we also observed an important contribution of ADAMTS1.Finally, we showed the impact of ADAMTS1 on IGFII-mediated IGF1R phosphorylation and cellular migration. Our results support a functional interaction between IGFBP2 and ADAMTS1 and suggest the need to evaluate post-translational modifications of IGFBP2 in glioma, in order to approach new therapies.

## INTRODUCTION

Recent findings are supporting the importance of post-translational modifications to the complex nature of the extracellular milieu. Among these processes, a profound understanding of extracellular proteolytic mechanisms remains essential, given its important implications during development, morphogenesis and tissue repair. Moreover, alterations in the expression of proteases has been strongly associated with pathological conditions, such as atherosclerosis and tumor progression[1]. Exceptional technological progresses are allowing a detailed analysis of the repertoire of proteolytic events in the extracellular milieu, illustrated by initiatives as the Degradome project[2]. Among the targets of proteolysis are growth factors, cell surface proteins and matrix components. The functional properties of these molecules depend on their integrity or specific fragmentation. Although post-translational modifications may alter the activation of key signaling pathways, still the assessment of prognosis of glioma patients, and neoplasias in general, is mainly based in clinic pathological factors.

Among the proteases involved in the modification of the extracellular microenvironment is ADAMTS1 (*a disintegrin and metalloprotease with thrombospondin motifs*), the first described member of the ADAMTS family of extracellular proteases[3]. Its catalytic activity has been reported on proteoglycans[4-6] and other extracellular components[7-9]. Although it was first shown to display anti-angiogenic properties[10], its role in tumorigenesis appears controversial. Various reports linked ADAMTS1 to the process of metastasis[11] and tumor growth[12-14] while its angiostatic capacities have also been reported[15] and associated with a new action in the process of tumor plasticity[16]. Still, the contribution of specific substrates of ADAMTS1 remains unknown. In line with our previous proteomic approach[9], here we used the Isotope-Coded Protein Label (ICPL) reagents[17] for the identification of ADAMTS1 substrates. In addition to confirming some of our previous findings, we were able to identify new putative substrates. Of special interest was the finding of IGFBP2 and other members of the IGFBP family that are known to be sensitive to proteolysis[18]. The regulatory capacities of IGFBPs for the bioavailability of Insulin-like Growth Factors (IGFs) appeared directly related with their proteolysis, although different roles during cancer progression have also been reported[19]. IGFBP2 has been correlated with increased malignancy in a variety of tumors (i.e. colon, breast, ovary, prostate, and more importantly, brain tumors[20]). These findings contradict the fact that IGFBPs were mainly considered as inhibitory factors of IGF actions, particularly IGFII with IGFBP2[19]. In fact, current therapeutic strategies are to inhibit IGF signaling pathways. Except for a few studies, no major attention has been directed to IGFBPs as the main binding proteins of these factors[21]. Importantly, alternative networks such as the signaling pathway integrin/ILK/NF-κB have been recently showed to be linked to IGFBP2 during glioma progression[22].

The use of IGFBP2 as a prognostic factor in identifying glioma requires further investigation. The results reported in this study provide a new area to be explored, proteolysis of IGFBP2 in gliomas.

## RESULTS

### Identification of IGFBP2 as a substrate of ADAMTS1

**Figure 1:**
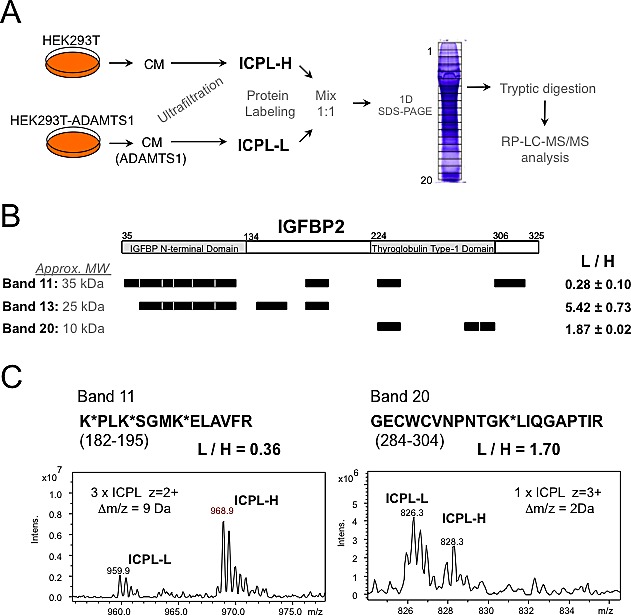
Proteomic approach for the identification of ADAMTS1 substrates (A) Workflow showing the steps followed to obtain labeled protein fragments and further spectrometry analyses (more details in Methods section). (B) Schematic representation of the IGFBP2 tryptic peptides identified in the LC-MS analysis on each of three different bands of the gel. According to molecular sizes, Band 11 would correspond to IGFBP2 full-length form, while Bands 13 and 20 would be N- and C-terminal fragments of the protein, respectively. The average and standard deviation of the measured ratios between the signals of Light and Heavy ICPL-labeled peptides in each band is shown. Aminoacid numbering is according to UnitProt Database (P18065). (C) Examples of the MS spectra for two labeled peptide pairs of IGFBP2. Left panel corresponds to a peptide for the analysis of the digest of Band 11, and right panel shows an example of a peptide from Band 20.

Protein from conditioned media from HEK293T cells, both parental and ADAMTS1-overexpressing, were obtained as for previous proteomic evaluations[9] (described in the Methods section). Our ICPL-based 1D-SDS LC-MS approach (Fig. 1A) allowed the identification of putative substrates of ADAMTS1. The validity of this methodology was confirmed by the identification of substrates formerly identified by other procedures, such as nidogen-1 and nidogen-2[9] (data not shown). In addition, new substrates were discovered, such as various members of the IGFBP family (IGFBP2, IGFBP4 and IGFBP5). It is known that proteolysis of these molecules is a required mechanisms for the release of IGFs[18]. Here, a detailed analysis of IGFBP2 proteolysis is presented (Fig. 1B-C). A number of peptides were identified in different 1D-SDS bands corresponding to full length IGFBP2 (Band 11) and its proteolytic fragments (Bands 13 and 20). The results showed a decrease of Band 11 peptides and an increase in the abundance of Band 13 and 20 peptides in the CM of ADAMTS1-overexpressing (labeled with light isotope) compared with parental cells (labeled with heavy isotope) (Fig. 1B). These observations demonstrate proteolysis of IGFBP2 by ADAMTS1. Two examples of the obtained MS spectra from specific peptides are showed (Fig. 1C).

**Figure 2:**
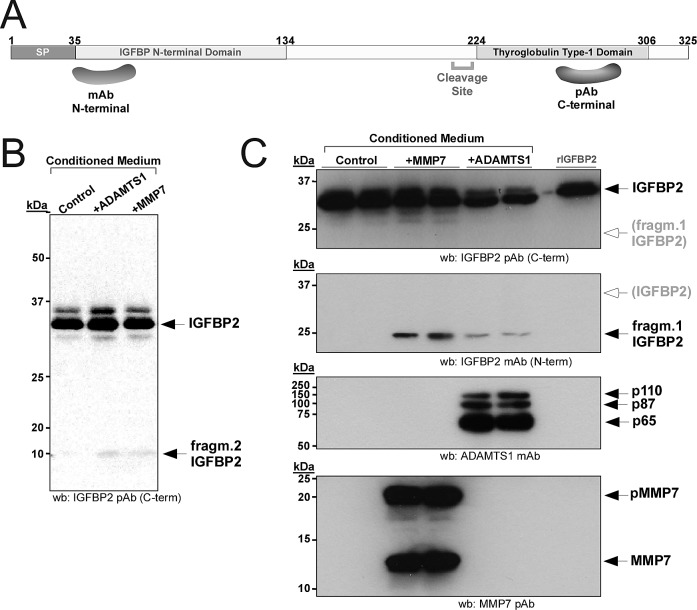
Proteolysis of IGFBP2 in HEK293T cells (A) Schematic representation of IGFBP2 protein, showing the fragments used for the generation of the antibodies employed in this study. (B, C) 24 h CM from control or transiently transfected cells with ADAMTS1 or MMP7 were collected and processed as indicated. Western-blots were performed with the indicated antibodies for each panel. All images in (C) belong to the same blot re-probed with indicated antibodies; white arrow/grey letters in 1^st^ and 2^nd^ panels points to the area where the other fragment would appear in 2^nd^ and 1^st^ panels respectively.

The verification of IGFBP2 cleavage in HEK293T cells was then analyzed by Western-blot. Metalloprotease MMP7, known to cleave IGFBP2, was used as a comparison to ADAMTS1 activity [23]. The over-expression of ADAMTS1 and MMP7 resulted in the appearance of a similar small fragment (named fragment 2, approx. 10 kDa), that was detected with an antibody against the C-end of IGFBP2 (Fig. 2B). This small fragment was always detected at very low levels, suggesting its low reactivity and/or instability. According to the proteomic data, an additional fragment of higher MW (approx. 25 kDa) was expected to be present, but it was not detected with this antibody for the C-terminus of IGFBP2. Therefore, another antibody raised against the N-end region of IGFBP2 was used. Importantly, this antibody showed the additional expected fragment (named fragment 1, approx. 25 kDa) that was generated in the presence of both ADAMTS1 and MMP7 (Fig. 2C, 2^nd^ panel). These observed fragments matched our ICPL results (fragment 1: Band 13, fragment 2: Band 20; Fig. 1B). In addition, these results emphasized the need to use a combination of antibodies in order to detect the different proteolytic fragments generated by the tested proteases.

### IGFBP2 is endogenously cleaved in glioma

**Figure 3:**
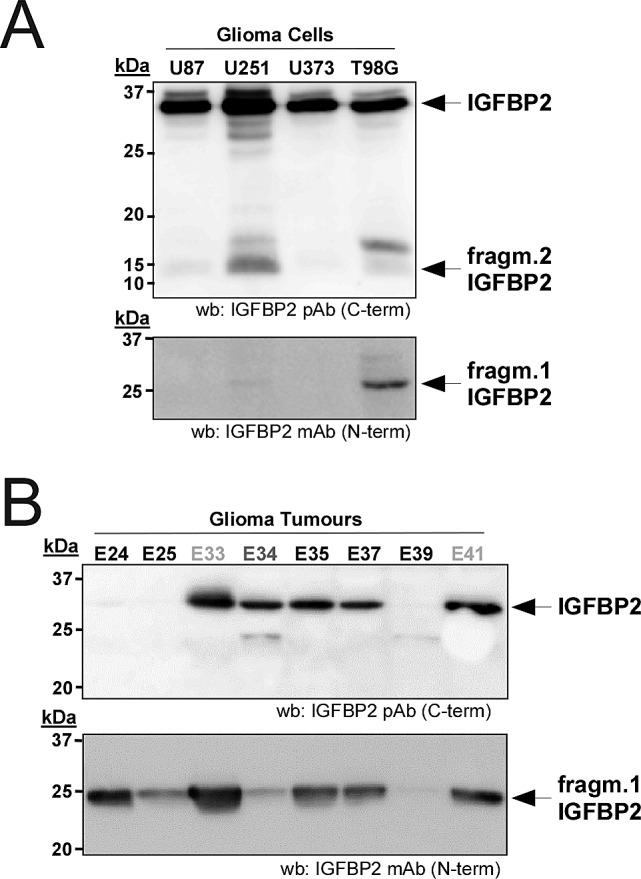
Relevance of IGFBP2 cleavage in gliomas (A) 24 h CM from glioma cells (U87, U251, U373 and T98G) were collected and processed as above. Blots were probed with the indicated antibodies for IGFBP2. (B) 100 ng of protein from human glioma tumors were analyzed by Western-blot with both IGFBP2 antibodies. The different samples are: Glioblastoma Multiforme (E24, E25, E35, E37, E39), Anaplasic Oligodendroglioma-Grade III (E34), and Difuse Fibrilar Astrocitoma-Grade II (E33, E41).

Although the expression of IGFBP2 has been extensively correlated with the progression of gliomas[24-26], its proteolytic pattern has not been evaluated nor its effects on the activation of IGFs pathways that are involved in neoplasia progression[27]. Our interests lead us to further investigate IGFBP2 proteolysis in human glioma cancer cell lines and tumor samples. Interestingly, all tested cells showed intact IGFBP2 and a small fragment (approx. 10-15 kDa) when analyzed with the C-terminal pAb. Furthermore, a larger fragment (approx. 25 kDa) was clearly detected in U251 and T98G cells with the N-terminal mAb for IGFBP2 (Fig. 3A). These patterns, detected using specific antibodies, were similar to the results observed in CM of HEK293T cells overexpressing ADAMTS1. This supports the existence of intrinsic specific proteolytic events in these glioma cells. Although this proteolytic outcome cannot be limited to the action of ADAMTS1, its expression in all the tested cell lines was confirmed by PCR analysis (Supplementary Fig. 1). The role of ADAMTS1 has been recently described in sarcoma and melanoma models[16], and the analysis of IGFBP2 proteolysis in EW7 and MUM2B cells (from sarcoma and melanoma, respectively) also showed similar patterns of proteolysis (Supplementary Fig. 2).

The evaluation of human glioma samples also revealed important findings. First, the use of the C-terminal antibody that identifies the full-length IGFBP2 protein and a shorter fragment showed that not all tumor samples presented the full-length protein (Fig. 3B, top panel). The smallest fragment was also not identified, in agreement with the cell culture observations that highlighted its low stability. Second, and more remarkable, all tested gliomas showed the cleaved form of IGFBP2 detected with the N-terminal antibody (Fig. 3B, bottom panel). The intensity of this cleaved IGFBP2 form and its ratio with the full-length molecule in every tumor sample showed different proportions, suggesting diverse proteolytic activities. Moreover, it is important to visualize this proteolytic process in the neoplasic context contributed by distinct cellular entities, such as inflammatory, endothelial and stromal cells, in addition to tumor cells.

**Figure 4:**
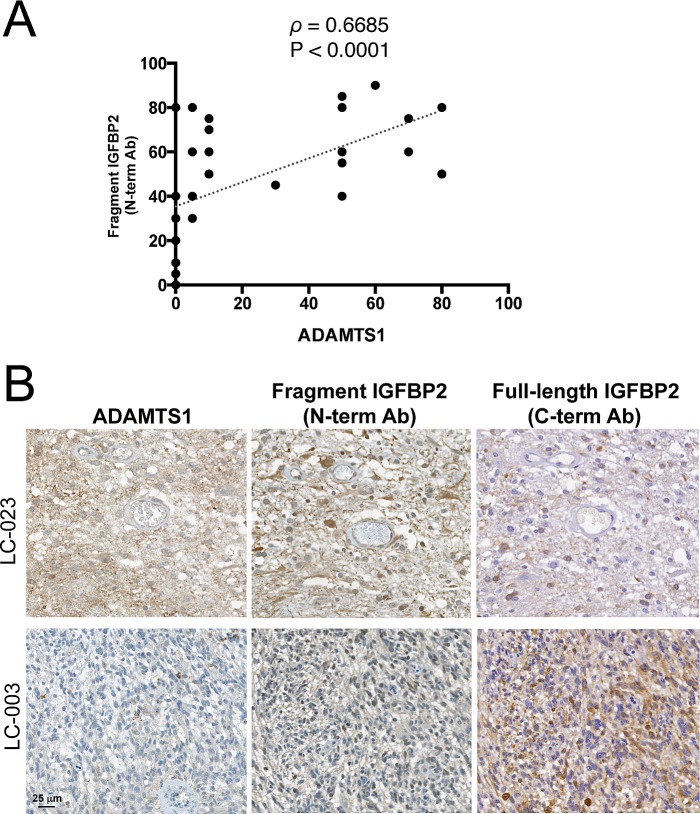
Correlation between ADAMTS1 and cleaved IGFBP2 in GBM (A) Graph showing the correlation between the expression of ADAMTS1 and those of the cleaved form of IGFBP2 (detected with N-term mAb) in tissue microarrays from a collection of human GBM samples. Spearman's correlation test was used to evaluate statistical significance; both correlation coefficient (ρ) and two-tailed significance (P) are shown. (B) Representative images from the tissue microarray are shown. Tumor 1 (LC-023) represents a case with high levels of ADAMTS1, high levels of cleaved IGFBP2 (with N-term mAb) and low levels of full-length IGFBP2 (with C-term pAb). Tumor 2 (LC-003) represents a case with low levels of ADAMTS1, low levels of cleaved IGFBP2 and high levels of full-length IGFBP2.

We also sought to determine by immunohistochemistry whether glioblastoma multiforme (GBM) cases, the most aggressive type of glioma, express ADAMTS1 and produce both the intact and the cleaved forms of IGFBP2. A collection of human GBM samples (up to 40 cases) arranged in a tissue microarray format was analyzed with ADAMTS1 and IGFBP2 antibodies as described in the Methods section. The levels of expression for these proteins were quantified (Supplementary Table 1). While the presence of ADAMTS1 was not detected in all GBM cases, 10 out of 33 samples had remarkably high ADAMTS1 levels. These evaluations also showed a wide expression of IGFBP2 protein in the majority of samples analyzed, in line with previous reports. Furthermore, these analyses detected the cleaved form of IGFBP2, showing a statistically significant correlation between the levels of this processed form with that of ADAMTS1 (Fig. 4A), which supports our hypothesis that ADAMTS1 cleaves IGFBP2 in glioma. Representative images of GBM cases with distinct levels of the protease and the IGFBP2 fragment are also reported here (Fig. 4B).

### Expression of IGFBP2 and ADAMTS1 display a direct correlation as bad prognostic markers of gliomas

**Figure 5:**
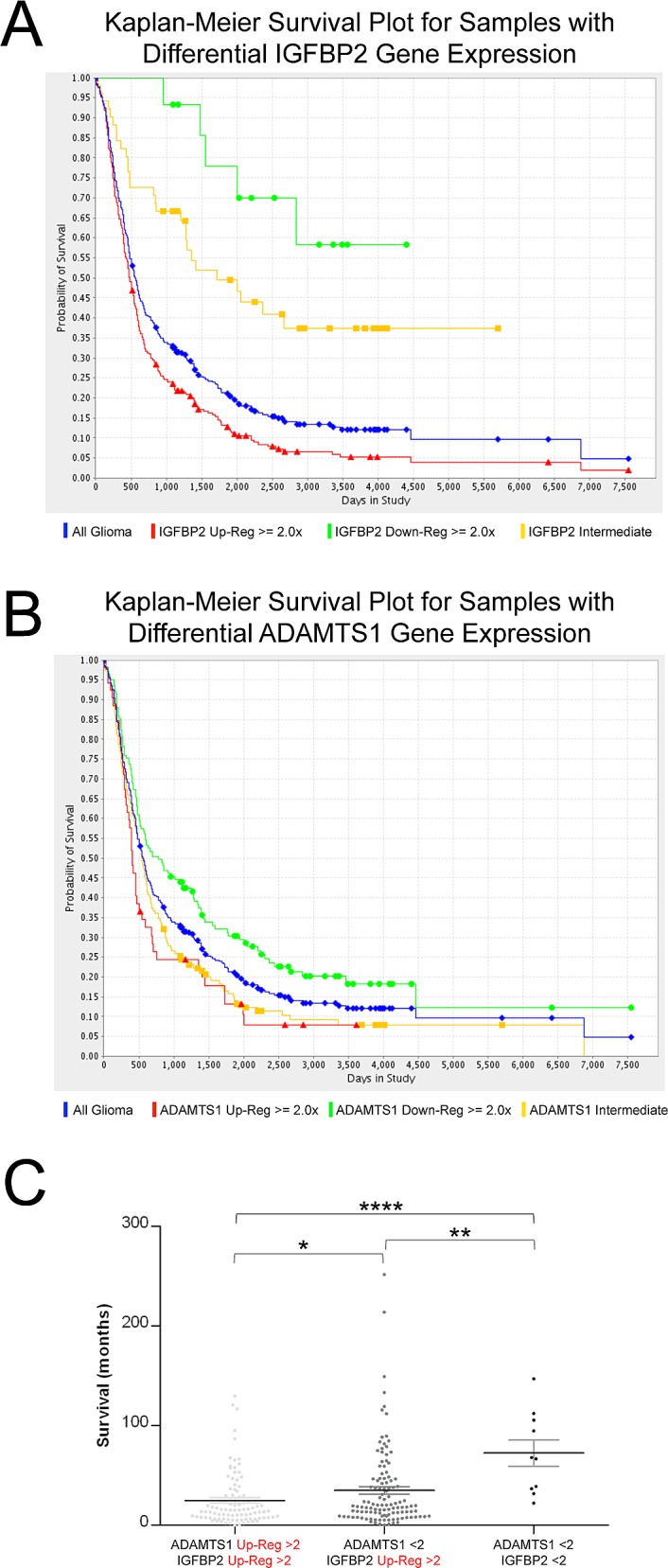
Evaluation of IGFBP2 and ADAMTS1 expression in the REMBRANDT database Representation of Kaplan-Meier Survival Plot for samples with differential expression of genes, IGFBP2 (A), and ADAMTS1 (B). Color code: Blue, all patients; Red, high expressors (>2); Yellow, intermediate expressors; Green, low expressors (<2). (C) Dot plot representing survival time of glioma patients from REMBRANDT database. Groups are: with up-regulated levels of both ADAMTS1 and IGFBP2 (>2), single up-regulated levels of IGFBP2 (>2), and without up-regulated levels of both genes. There were not samples with single up-regulated levels of ADAMTS1 (>2). Statistical analysis was approached by Mann Whitney Test (*, *p<0.05*; **, *p<0.005*; ****, *p<0.0001*).

IGFBP2 has been proposed by various groups as a prognostic maker of tumor grading of gliomas[24-26]. To further look into this prospect, we decided to closely analyze the National Cancer Institute public database REMBRANDT (REpository for Molecular BRAin Neoplasia DaTa). These studies confirmed the relevance of this molecule in gliomas. Here, a survival curve of glioma patients in relationship to their IGFBP2 gene expression data is shown (Fig. 5A); there are significant differences among high-, low- and intermediate-expressors (*Log-rank p-value=6.564E-7, Up-regul. vs. Down-regul.; Log-rank p-value=1.28E-8, Up-regul. vs. Interm.*). A detailed statistical analysis is reported in Supplementary Fig. 3. In a similar manner, this database allowed us to evaluate the survival plot according to expression levels of ADAMTS1 (Fig. 5B). Although the differences among high and low-expressors of ADAMTS1 are not as significant as for IGFBP2, they still displayed statistical relevance (data in Supplementary Fig. 3). Based on our experimental results shown above, we decided to further analyze this database to determine the frequency of co-expression of our molecules of interest, ADAMTS1 and its proteolytic target IGFBP2, and to determine their potential contribution to the evolution of the disease (Supplementary Table 2). The survival of patients were evaluated and compared according to the expression of both ADAMTS1 and IGFBP2 (Fig. 5C). Interestingly, this analysis revealed that the group of patients with glioma that have up-regulated levels of both ADAMTS1 and IGFBP2 showed a shorter survival time, with significant differences to patients with up-regulated levels of IGFBP2 (*p-value=0.0352*), and to patients without up-regulation of any of these genes (*p-value<0.0001*). Finally, co-expression of both molecules was evaluated in *Oncomine.org* database showing relevant results in the Bredel study (*correlation index=0.658*) that distinguishes different grade gliomas and normal brain (Supplementary Figure 4).

These results support the hypothesis that the presence of the protease ADAMTS1 may affect the integrity of IGFBP2 in a vast number of specimens, consistent with the Western-Blot results. The high number of samples in these databases emphasizes the importance of the analysis.

### Actions of ADAMTS1 on IGFBP2 affect IGFII signaling

**Figure 6:**
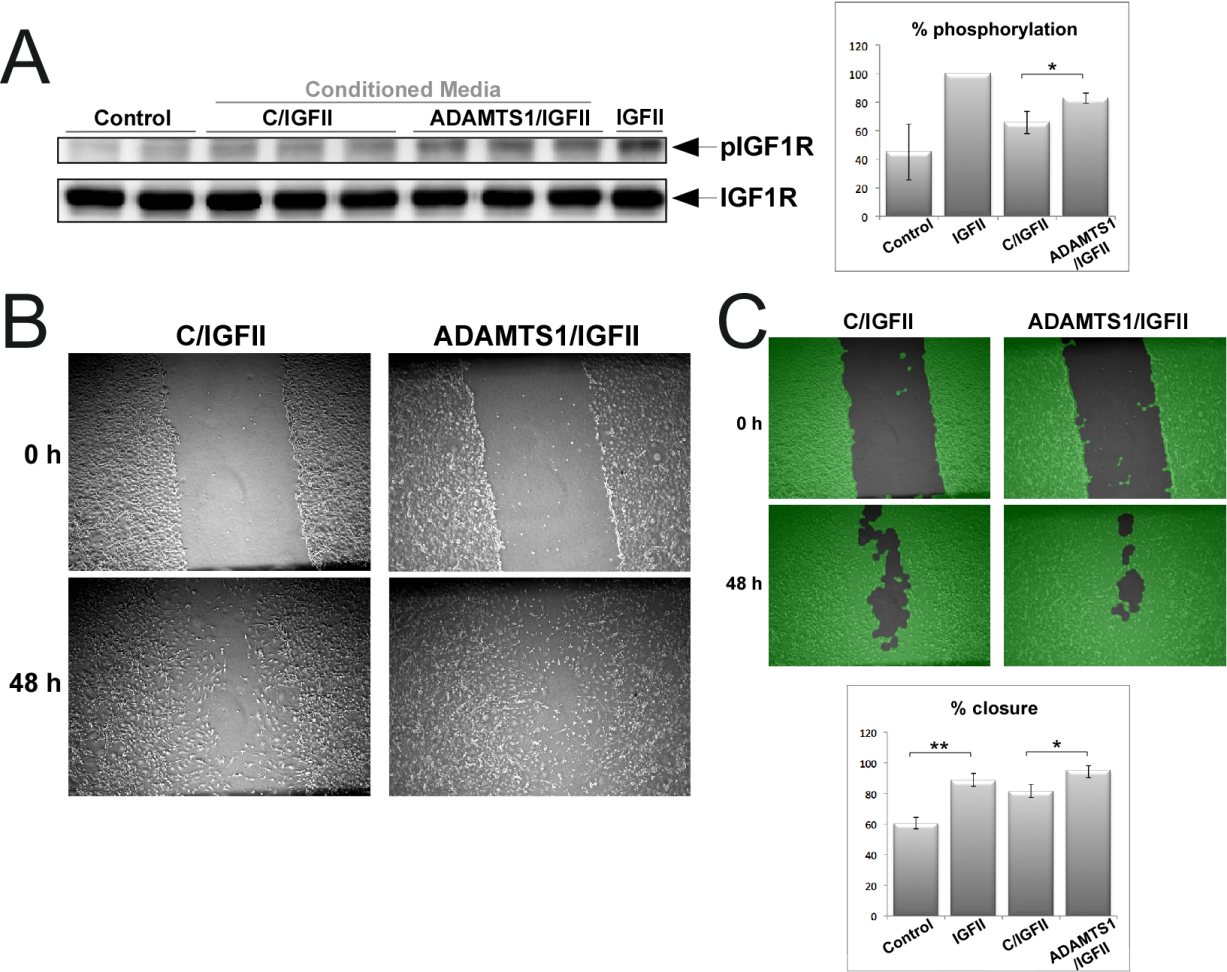
Effects of ADAMTS1 in IGFII signaling For these assays, CM (from control and ADAMTS1-overexpressing cells) was previously incubated with 5 ng/ml rIGFII (R&D) during 30 min. (A) 3T3-IGF1R mouse fibroblasts (overexpressing IGF1R) were kept without serum overnight to down-regulate basal phosphorylation levels. Then, cells were incubated during 10 min with the specified CM. Cell lysates were harvested with RIPA buffer with protease inhibitors, and resolved for further Western-blotting analysis. Blots were probed with specified antibodies for the phosphorylated form of IGF1R and for total protein. Graph shows % levels of pIGF1R. (B) Representative images of the wound-healing assay performed on a monolayer of confluent 3T3-IGF1R cells. (C) For proper quantification, images were processed by the WimScratch platform (www.wimasis.com). Pictures in green show the computerized processing of images resulted from this platform. Graph shows the results of triplicate wells of at least 2-3 different experiments. For both graphs, statistics significance was approached by T-test analysis (*, p<0,05; **, p<0,005).

Our next goal was to understand the effects of IGFBP2 cleavage on the bioavailability of IGFs. The phosphorylation of IGF1R under different conditions was first assessed in a cell culture scenario. 3T3-IGF1R cells (a generous gift from Dr. A. Ullrich) were treated with 24 h CM from control and ADAMTS1-overexpresor cells, which were previously incubated for 30 minutes with IGFII. We hypothesize that ADAMTS1 would provoke an increased release of IGFII from proteolysed IGFBP2; therefore, an increase of free IGFII in the presence of ADAMTS1 would be observed (Supplementary Fig. 5). The analysis of IGF1R phosphorylation (pIGF1R) showed increased levels in the presence of ADAMTS1 based on the augmented bioavailability of the ligand IGFII (Fig. 6A). The quantification of these signals showed significant differences (*p=0.0266, Student's t-test*) (Fig. 6A).

As a second approach, we performed wound-healing assays of 3T3-IGF1R cells under similar conditions, using CM from control and ADAMTS1-overexpresor cells previously incubated with IGFII. As shown, the presence of ADAMTS1 provoked a faster closure of the wound (Fig. 6B). Importantly, as a quantitative tool, an automated image analysis that provides unbiased results, the WimScratch platform (www.wimasis.com), was used. This analysis also reported a significant difference (p=0.040) (Fig. 6C). These results confirmed our hypothesis that IGFII release after cleavage of IGFBP2 is important for the activation of IGF1R, which affects its phosphorylation status and migratory activity.

## DISCUSSION

It is fully accepted that the elucidation of the oncogenic pathways driving tumor progression would help to postulate new therapeutic targets. While genomic studies have strongly suggested the expression of IGFBP2 to be used as a bad prognosis factor in glioma, our studies support for the first time, at least to our knowledge, the need to analyze the cleavage of IGFBP2 protein in human glioma specimens.

By a combination of proteomic studies and *in vivo* biochemical analyses, we revealed the induced proteolysis of IGFBP2 by ADAMTS1. Further evaluation of glioma samples, mostly focused in GBM, and of qualified databases, verified the presence of IGFBP2 proteolysis in gliomas and revealed a significant correlation between ADAMTS1 and IGFBP2. From a technical perspective, we demonstrated the value of ICPL technology to approach the identification of substrates of proteases in a biological setting. At this point, further advances in proteomics are required in order for it to be adapted to clinical applications as suggested in our current study. While we report here the importance of analyzing protein integrity, in addition to gene expression, we also realize the difficulty of carrying out these studies given the difficulty to obtain these valuable samples.

Our study showed endogenous cleavage of IGFBP2 in different tumor cell lines and, more importantly, in glioma samples by Western-blot and immunohistochemistry. It highlights the importance of post-translational modifications on IGFBP2 to determine its function. The fact that all human glioma samples displayed proteolysis, some of them at very high rates, suggests the need to re-evaluate the claims of oncogenic attributes of IGFBP2. Our results emphasize the possibility that there is a concomitant requirement of proteolysis to display such pro-tumorigenic properties. In fact, the extrapolation of our phosphorylation and migration assays supports that the proteolysis of IGFBP2 will be accompanied by increased bioavailability of IGFs in gliomas. Consequently, the activation of IGFs-dependent signaling pathways would have major consequences for tumor cell proliferation, migration and invasiveness. In line with our findings, a recent report showed the inhibition of tumor growth by expressing a protease-resistant IGFBP4 form[21]. At this point, we need to consider that the pro-tumorigenic actions of IGFBP2 are due to the fact that it is mainly found in a cleaved form. Although the knowledge of its levels of expression is still important, the revelation of its proteolytic status would provide a more realistic proof of functionality. These results raise the possibility of using specific metalloprotease inhibitors to block IGFBP proteolysis, probably in conjunction with additional therapies as the direct blockade of IGFs pathways. More profound research needs to be performed.

Importantly, it is necessary to highlight recent advances that suggest alternative mechanisms of action for IGFBP2, as it is an integrin/integrin-linked kinase/NF-κB pathway[22]. It is known that the integrin binding activity motif of IGFBP2 is found at its C-terminus end, but the influence of proteolysis and potential activity of the generated fragments has not been addressed.

So far, the cleavage of IGFBPs has been attributed to several MMPs although other proteases also appear to cleave these molecules, such as ADAMs, PAPP-A, and others[18]. Our studies do not exclude other proteases to be the cause of IGFBP2 cleavage in gliomas and, in fact, we consider this a relevant field of study. Nevertheless, the analysis of the REMBRANDT database did not provide a relevant correlation with closely related proteases to ADAMTS1, such as ADAMTS4 and ADAMTS5 (Supplementary Fig. 3). We support the hypothesis that the presence of a specific protease affects IGFBP2 integrity in a vast number of specimens, which is consistent with our Western blot and immunohistochemistry results, and would add relevance to assess prognosis. The high number of samples in these databases emphasizes the statistical significance of the analysis.

In our *in vivo* approaches, we confirmed the activity of MMP7 and included the contribution of ADAMTS1 using a combination of proteomic techniques. With regards to the ADAMTS family of extracellular proteases, we added a new function, cleavage of IGFBP2. Previous reports have already associated ADAMTS proteases with glioma progression, mainly by their proteolytic activity on the proteoglycans brevican and versican, as implicated in the biology of gliomas[28-30]. In addition, ADAMTS1 has been implicated in phenomena of tumor plasticity for the acquisition of endothelial-related features in sarcoma and melanoma[16]. Interestingly, events of tumor vascularization by endothelial differentiation of glioblastoma cells have also been recently described as highly relevant[31,32]. On the other hand, it has been reported that IGFBP2 promotes the survival of glioma tumor stem cells[33], a hypothetical origin of trans-differentiation phenomenon. In accordance with these observations, it appears that studies regarding a contribution of the partnership between ADAMTS1 and IGFBP2 are important to pursue.

In conclusion, our results strongly suggest the need to re-evaluate current therapies against glioma. Although our perspective does not minimize the relevance of IGF-targeted therapies, we propose that the analysis of IGFBP2 proteolysis will provide major contributions to properly modify or implement current strategies, such as the value of specific proteolytic inhibitors or even the use of optimized IGF-binding partners to prevent an excessive bioavaibility of IGFs to tumor cells. Finally, the most recent perspectives that remark the use of personalized medicine would require a closer attention to post-translational modifications.

## METHODS

### Cell culture, transfections and treatments

Cell lines used are: HEK293T from human embryonic kidney; U87, U251, U373, and T98G from human gliomas; and 3T3-IGF1R (kindly provided by Dr. Ullrich), modified from 3T3 fibroblasts. All cells were maintained at 37 ºC, 5% CO2, in media supplemented with 10% fetal bovine serum (FBS), 1% penicillin-streptomycin and 1% 200 mM L-glutamine. Transient transfections with ADAMTS1 and MMP7 expression vectors were performed with Fugene 6.0 reagent (Promega) according to manufacturer's instructions. For phosphorylation assays, 3T3-IGF1R mouse fibroblasts (overexpressing IGF1R) were kept without serum overnight to down-regulate basal phosphorylation levels. Prior to cell incubation, conditioned media (CM) (from control and ADAMTS1-overexpressing cells) were incubated with 5 ng/ml rIGFII (R&D) for 30 min. Then, 3T3-IGF1R cells were incubated for 10 min with this specified CM. Cell lysates were harvested with RIPA buffer with protease inhibitors, and resolved for further Western-blotting analysis. For wound healing assays, 3T3-IGF1R cells were maintained to obtain monolayer of confluent cells. After a transversal wound was generated, cells were washed twice and incubated with specific CM for 48 h. Pictures were taken at the specified times. For proper quantification, images were processed by the WimScratch platform (www.wimasis.com).

### Sample preparation for ICPL analysis

Parental and ADAMTS1-overexpressing HEK293T cells were cultured as previously described for DIGE screening[9]. 24 h CM was collected from each cell culture (Workflow in Fig. 1A). Proteins in the CM were first concentrated by ultrafiltration, and then labeled with heavy (parental) and light (ADAMTS1) forms of SERVA ICPL kit. Labeled proteins were mixed 1:1 and fractionated by 1D-SDS-PAGE. Gel lane was cut in twenty slices, which were then subjected to tryptic digestion. Each digest was analyzed by Nano Reverse Phase-Liquid Chromatography coupled to electrospray Mass Spectrometry (RP-LC-MS/MS) on an Ion-Trap instrument (Bruker Esquire, Bremen).

### Human glioma tumors

Human glioma tumors were properly obtained from the Oncology Unit of the Vall d'Hebron Research University Hospital and from the Institute of Neuropathology-Hospital Universitari Bellvitge (tissue microarray GBMs) according to the World Medical Association Declaration of Helsinki. Tumor data from the REMBRANDT (Repository of Molecular Brain Neoplasia Data) web application database were used according to their instructions (http://caintegrator-info.nci.nih.gov/rembrandt).

### Immunoblot and immunohistochemical analyses

For evaluation of cell cultures by Western blot, CM was clarified and concentrated with StrataClean resin (Stratagene) as previously described [9]. Total protein from tumor samples and cell lysates was extracted with RIPA buffer containing 1 mM PMSF, 1 μg/ml aprotinin and 10 μg/ml leupeptin. Proteins were resolved by SDS-PAGE and transferred to nitrocellulose (Schleicher & Schuel) or PVDF (BioRad) membranes. Membranes were blocked with 5% low-fat milk and incubated with the following primary antibodies: monoclonal mouse anti-human ADAMTS1 (clone 5D4E11B5, kindly provided by Dr. M.L. Iruela-Arispe); polyclonal goat anti-human C-terminus IGFBP2 (sc-6001 (C-18), Santa Cruz Biotechnology); monoclonal mouse anti-human N-terminus IGFBP2 (sc-25285 (C-10), Santa Cruz Biotechnology); monoclonal mouse anti-human MMP7 (IM71, Calbiochem); polyclonal rabbit anti-human IGF1R (phosphorylation sites Tyr^1158/1162/1163^, PS1009, Calbiochem); polyclonal rabbit anti-human IGF-1Rβ (sc-713, Santa Cruz Biotechnology). After incubation with the appropriate secondary peroxidase-conjugated antibodies, signal was detected with the SuperSignal West Dura Chemiluminescence Kit (Pierce). ImageJ Software (NIH) was used for quantification of images. Significance was approached by T-test statistical analysis. For immunohistochemical staining, tissue microarray samples were stained with the following primary antibodies: polyclonal rabbit anti-human ADAMTS1 (H-60, sc-25581, Santa Cruz Biotechnology) and antibodies for IGFBP2 forms described above.

### Statistical analyses

Distinct statistical approaches used throughout the manuscript are specifically indicated in the figure legends. These analyses were carried out using the GraphPad Prism 6 Software, unless otherwise indicated.

## SUPPLEMENTARY FIGURES



## SUPPLEMENTARY TABLE



## SUPPLEMENTARY TABLE



## References

[1] Werb Z (1997). ECM and cell surface proteolysis: Regulating cellular ecology. Cell.

[2] Lopez-Otin C, Overall CM (2002). Protease degradomics: a new challenge for proteomics. Nat Rev Mol Cell Biol.

[3] Apte SS (2009). A disintegrin-like and metalloprotease (reprolysin-type) with thrombospondin type 1 motif (ADAMTS) superfamily: functions and mechanisms. J Biol Chem.

[4] Sandy JD, Westling J, Kenagy RD, Iruela-Arispe ML, Verscharen C, Rodriguez-Manzaneque JC (2001). Versican V1 proteolysis in human aorta in vivo occurs at the Glu441-Ala442 bond, a site which is cleaved by recombinant ADAMTS-1 and ADAMTS-4. J Biol Chem.

[5] Rodriguez-Manzaneque JC, Carpizo D, Plaza-Calonge Mdel C, Torres-Collado AX, Thai SN, Simons M (2009). Cleavage of syndecan-4 by ADAMTS1 provokes defects in adhesion. Int J Biochem Cell Biol.

[6] Rodriguez-Manzaneque JC, Westling J, Thai SN, Luque A, Knauper V, Murphy G (2002). ADAMTS1 cleaves aggrecan at multiple sites and is differentially inhibited by metalloproteinase inhibitors. Biochem Biophys Res Commun.

[7] Esselens C, Malapeira J, Colome N, Casal C, Rodriguez-Manzaneque JC, Canals F (2010). The cleavage of semaphorin 3C induced by ADAMTS1 promotes cell migration. J Biol Chem.

[8] Torres-Collado AX, Kisiel W, Iruela-Arispe ML, Rodriguez-Manzaneque JC (2006). ADAMTS1 interacts with, cleaves, and modifies the extracellular location of the matrix inhibitor tissue factor pathway inhibitor-2. J Biol Chem.

[9] Canals F, Colome N, Ferrer C, Plaza-Calonge Mdel C, Rodriguez-Manzaneque JC (2006). Identification of substrates of the extracellular protease ADAMTS1 by DIGE proteomic analysis. Proteomics.

[10] Vazquez F, Hastings G, Ortega MA, Lane TF, Oikemus S, Lombardo M (1999). METH-1, a human ortholog of ADAMTS-1, and METH-2 are members of a new family of proteins with angio-inhibitory activity. J Biol Chem.

[11] Liu YJ, Xu Y, Yu Q (2006). Full-length ADAMTS-1 and the ADAMTS-1 fragments display pro- and antimetastatic activity, respectively. Oncogene.

[12] Ricciardelli C, Frewin KM, Tan ID, Williams ED, Opeskin K, Pritchard MA The ADAMTS1 Protease Gene Is Required for Mammary Tumor Growth and Metastasis. Am J Pathol.

[13] Rocks N, Paulissen G, Quesada-Calvo F, Munaut C, Gonzalez ML, Gueders M (2008). ADAMTS-1 metalloproteinase promotes tumor development through the induction of a stromal reaction in vivo. Cancer Res.

[14] Martino-Echarri E, Fernández-Rodríguez R, Rodríguez-Baena FJ, Barrientos-Durán A, Torres-Collado AX, Plaza-Calonge M, del C (2013 Nov 15). Contribution of ADAMTS1 as a tumor suppressor gene in human breast carcinoma. Linking its tumor inhibitory properties to its proteolytic activity on nidogen-1 and nidogen-2. Int J Cancer.

[15] Reynolds LE, Watson AR, Baker M, Jones TA, D'Amico G, Robinson SD (2010 Jun 10). Tumour angiogenesis is reduced in the Tc1 mouse model of Down's syndrome. Nature.

[16] Casal C, Torres-Collado AX, Plaza-Calonge Mdel C, Martino-Echarri E, Ramon YCS, Rojo F (2010). ADAMTS1 contributes to the acquisition of an endothelial-like phenotype in plastic tumor cells. Cancer Res.

[17] Schmidt A, Kellermann J, Lottspeich F (2005 Jan). A novel strategy for quantitative proteomics using isotope-coded protein labels. Proteomics.

[18] Bunn RC, Fowlkes JL (2003). Insulin-like growth factor binding protein proteolysis. Trends Endocrinol Metab.

[19] Firth SM, Baxter RC (2002). Cellular Actions of the Insulin-Like Growth Factor Binding Proteins. Endocr Rev.

[20] Fukushima T, Kataoka H (2007). Roles of insulin-like growth factor binding protein-2 (IGFBP-2) in glioblastoma. Anticancer Res. 2007/11/01 ed.

[21] Ryan AJ, Napoletano S, Fitzpatrick PA, Currid CA, O'Sullivan NC, Harmey JH (2009). Expression of a protease-resistant insulin-like growth factor-binding protein-4 inhibits tumour growth in a murine model of breast cancer. Br J Cancer.

[22] Holmes KM, Annala M, Chua CYX, Dunlap SM, Liu Y, Hugen N (2012 Feb 28). Insulin-like growth factor-binding protein 2-driven glioma progression is prevented by blocking a clinically significant integrin, integrin-linked kinase, and NF-κB network. Proc Natl Acad Sci U S A.

[23] Nakamura M, Miyamoto S, Maeda H, Ishii G, Hasebe T, Chiba T (2005). Matrix metalloproteinase-7 degrades all insulin-like growth factor binding proteins and facilitates insulin-like growth factor bioavailability. Biochem Biophys Res Commun.

[24] Elmlinger MW, Deininger MH, Schuett BS, Meyermann R, Duffner F, Grote EH (2001). In vivo expression of insulin-like growth factor-binding protein-2 in human gliomas increases with the tumor grade. Endocrinology.

[25] Lin Y, Jiang T, Zhou K, Xu L, Chen B, Li G (2009). Plasma IGFBP-2 levels predict clinical outcomes of patients with high-grade gliomas. Neuro Oncol.

[26] Wang H, Shen W, Huang H, Hu L, Ramdas L, Zhou YH (2003). Insulin-like growth factor binding protein 2 enhances glioblastoma invasion by activating invasion-enhancing genes. Cancer Res. 2003/08/09 ed.

[27] Pollak M (2008). Insulin and insulin-like growth factor signalling in neoplasia. Nat Rev Cancer.

[28] Matthews RT, Gary SC, Zerillo C, Pratta M, Solomon K, Arner EC (2000). Brain-enriched hyaluronan binding (BEHAB)/Brevican cleavage in a glioma cell line is mediated by a disintegrin and metalloproteinase with thrombospondin motifs (ADAMTS) family member. J Biol Chem.

[29] Varga I, Hutoczki G, Szemcsak CD, Zahuczky G, Toth J, Adamecz Z (2011). Brevican, Neurocan, Tenascin-C and Versican are Mainly Responsible for the Invasiveness of Low-Grade Astrocytoma. Pathol Oncol Res.

[30] Viapiano MS, Hockfield S, Matthews RT (2008). BEHAB/brevican requires ADAMTS-mediated proteolytic cleavage to promote glioma invasion. J Neurooncol.

[31] Ricci-Vitiani L, Pallini R, Biffoni M, Todaro M, Invernici G, Cenci T (2010). Tumour vascularization via endothelial differentiation of glioblastoma stem-like cells. Nature.

[32] Wang R, Chadalavada K, Wilshire J, Kowalik U, Hovinga KE, Geber A (2010). Glioblastoma stem-like cells give rise to tumour endothelium. Nature.

[33] Hsieh D, Hsieh A, Stea B, Ellsworth R (2010 Jun 25). IGFBP2 promotes glioma tumor stem cell expansion and survival. Biochem Biophys Res Commun.

