# Supplementary material for: Relevance of IGFBP2 proteolysis in glioma and contribution of the extracellular protease ADAMTS1
Source: Oncotarget. 2014 May 26;5(12):4295–304. doi: 10.18632/oncotarget.2009 (PMC4147324; doi:10.18632/oncotarget.2009)
Supplement: Supplementary file 1 [file oncotarget-05-4295-s001.pdf]

Relevance of IGFBP2 proteolysis in glioma and contribution of the extracellular protease ADAMTS1

Supplementary Material

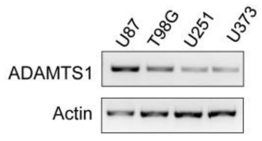

Supplementary Figure 1: PCR analysis for ADAMTS1 and actin expression in glioma cell lines.

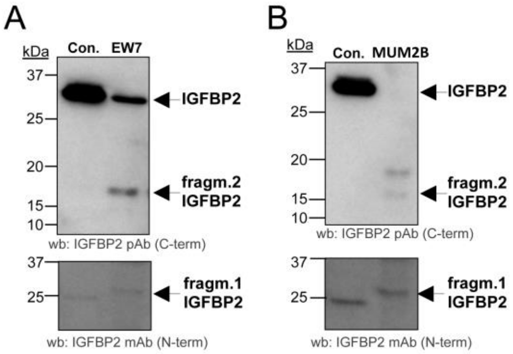

Supplementary Figure 2: 24h CM from Control (Con.) and EW7 (Panel A) or MUM2B (Panel B) cell lines (sarcoma and melanoma respectively) were analysed by Western blot with the indicated antibodies. Control CM was obtained from HEK293 overexpressing cells as indicated in the main text.

A

Statistical Report from REMBRANDT Database

	IGFBP2	ADAMTS1	ADAMTS4	ADAMTS5
**Number of samples in group:**				
Up-Regulated:	277	52		209
Down-Regulated:	15	141	30	5
Intermediate:	51	150	313	129
**Log-rank p-value(for significance of difference of survival between group of samples)**				
Up-Regulated vs. Intermediate:	1.28E-8	0.437612		0.014570
Up-regulated vs. Down-Regulated:	6.56E-7	0.002769		0.291714
Down-Regulated vs. Intermediate	0.068355	0.001453	0.249203	0.629577
Up-Regulated vs. all other samples:	0.0	0.040033		0.010829
Down-Regulated vs. all other samples:	1.83E-5	2.99E-4	0.249203	0.404312
Intermediate vs. all other samples:	4.79E-7	0.021082	0.249203	0.017698

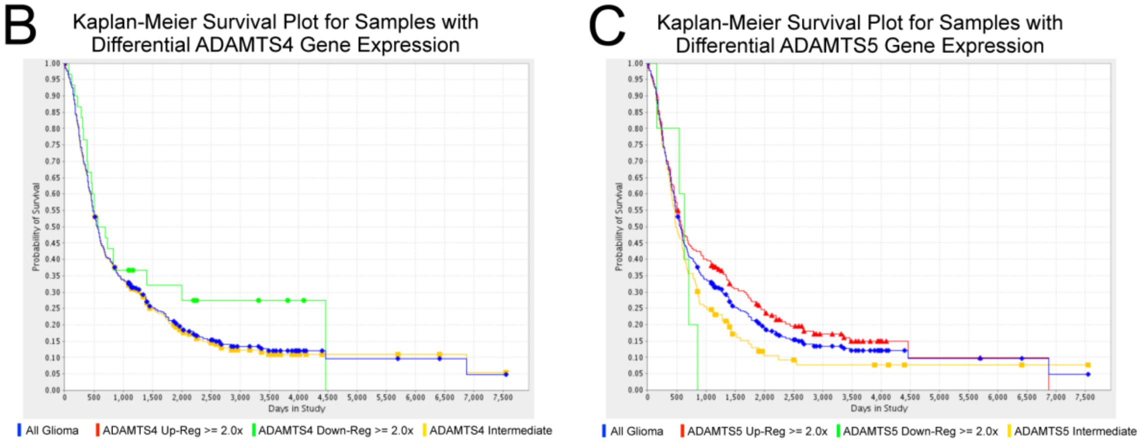

Supplementary Figure 3: Statistical Reports and Survival plots from REMBRANDT Database. (A) Summary of statistical analysis of the following genes: IGFBP2, ADAMTS1, ADAMTS4 and ADAMTS5. (B, C) Representation of Kaplan-Meier Survival Plot for samples with differential expression of ADAMTS4 (B) and ADAMTS5 (C).

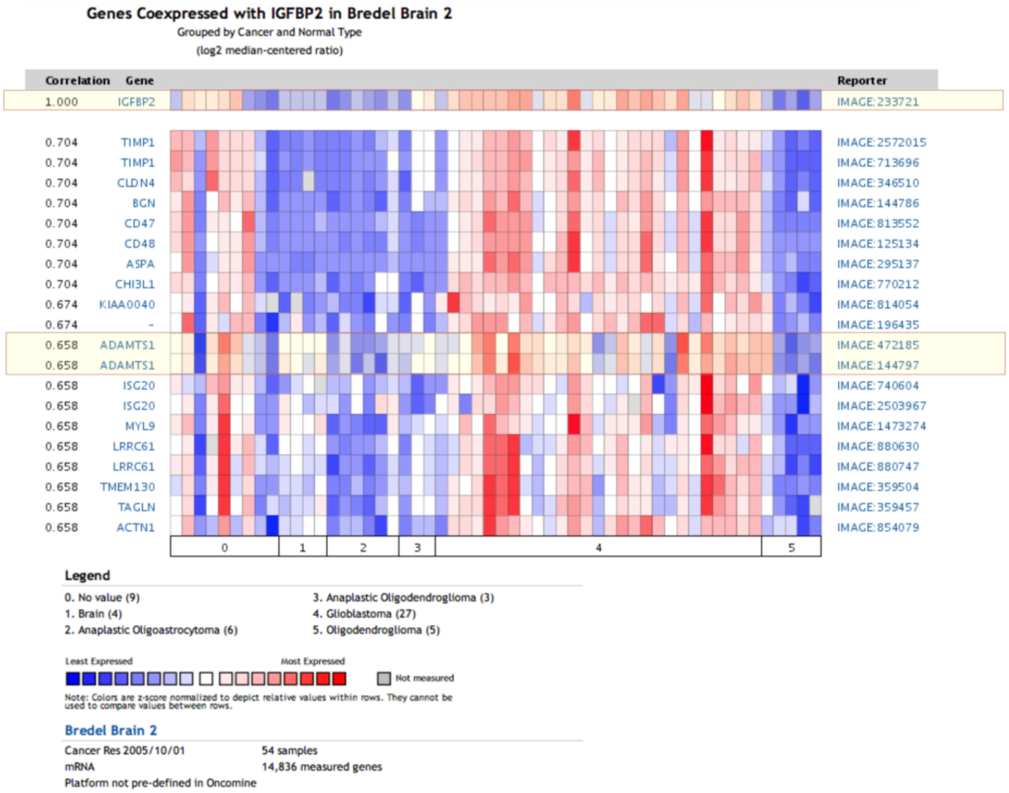

Supplementary Figure 4: Oncomine (oncomine.org) results for Bredel study showing a correlation between IGFBP2 and ADAMTS1 expression in glioma specimens (*correlation index=0.658*). This correlation value is an approximate correlation derived from average linkage hierarchical clustering. It is derived from the correlation value of the node at which the query gene (IGFBP2) and the target gene (ADAMTS1) were joined. The node correlation value is computed as the average of all pair-wise correlations among genes included at the node. Coexpression results are generated for 10 reporters per node where the correlation is 0.5 or higher. The heat map orders genes by their correlation value.

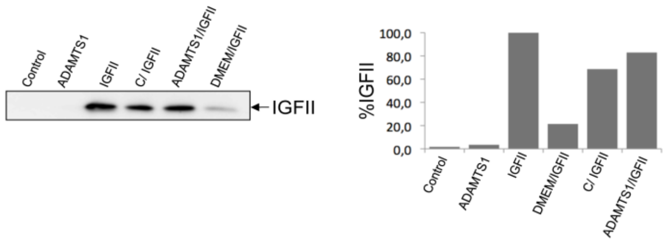

Supplementary Figure 5: WB analysis of IGFII presence under different conditions as used in Figure 5. Mainly, 5 ng/ml of rIGFII (from R&D) were pre-incubated during 30 min with 24 h CM from parental and ADAMTS1-overexpressor cells (lanes *C/IGFII* and *ADAMTS1/IGFII* respectively). As controls, similar IGFII amount was directly resolved by SDS-PAGE (lane *IGFII*), and incubated with fresh DMEM (lane *DMEM/IGFII*).
Additional controls are: 24 h CM from cells without IGFII pre-incubation.
Quantification of IGFII signal is performed with ImageJ software. % is calculated considering lane *IGFII* as 100%.
